# Use of Olive Pulp for Gestating Iberian Sow Feeding: Influence on Performance, Health Status Indicators, and Fecal Microbiota

**DOI:** 10.3390/ani12223178

**Published:** 2022-11-17

**Authors:** Cristian Jesús Sánchez, Belén Barrero-Domínguez, Silvia Martínez-Miró, Josefa Madrid, Alberto Baños, María Arántzazu Aguinaga, Silvia López, Fuensanta Hernández

**Affiliations:** 1 Department of Animal Production, Faculty of Veterinary Science, Regional Campus of International Excellence “Mare Nostrum”, University of Murcia, 30100 Murcia, Murcia, Spain; 2 Dcoop Sociedad Cooperativa Andaluza, Carretera Córdoba S/N, 29200 Antequera, Málaga, Spain; 3 DMC Research Center, Camino de Jayena, 82, 18620 Alhendín, Granada, Spain

**Keywords:** olive by-product, antioxidant status, intestinal health, fibrous feeds, local resources

## Abstract

**Simple Summary:**

Reutilization of olive oil by-products in animal feeding could be considered an alternative for attenuating the environmental problems derived from their cumulation. Olive pulp along with other olive oil by-products are characterized by high fiber and bioactive compounds content, which can be interesting in feeding pregnant sows. The objective of this study was to determine the influence of olive pulp inclusion in Iberian sow’s diet on performance, immunoglobulin levels and serum parameters, antioxidant capacity, and fecal microbiota. The experience showed a positive effect of olive pulp inclusion on the sow’s fecal bacterial counts and antioxidant status, but no effects were found on the rest of the parameters studied. These results are promising from a nutritional point of view, as olive pulp can be considered an ingredient to include in Iberian sow’s formulation program. Nonetheless, more research is needed to be able to consider olive pulp in Iberian sow’s diet, especially from the sustainability aspect, since both productions coexist.

**Abstract:**

Olive pulp (*Olea europaea*) inclusion in the diet of Iberian sows (*Sus scrofa*) is interesting due to fiber and bioactive compounds content and because both productions are located in the same area. The aim of this trial was to study the effect of olive pulp inclusion at 100 g/kg in Iberian sow’s diet on performance, immunoglobulin and serum parameters, antioxidant status, and fecal microbiota. Forty multiparous Iberian sows (body weight (BW) = 149.6 ± 20.2 kg) were assigned either a control diet (CON) or an experimental diet (PUL) with olive pulp at 100 g/kg. The BW and backfat thickness in sows were measured at post-insemination days 42 and 107, and litter performance was measured on the farrowing day. Blood and fecal samples were collected at gestation day 107. In piglets, blood was sampled when they were 10 days old for immunoglobulin analysis. Albumin, total protein, triglyceride, creatinine, urea, glucose, and Trolox equivalent antioxidant capacity in serum were higher (*p* < 0.05) in PUL sows than in CON sows. The *Enterobacteriaceae*, *Bifidobacterium* spp., and *Lactobacillus* spp. fecal counts were increased (*p* < 0.05) with olive pulp supplementation compared with the CON sow group. Olive pulp added to gestating Iberian sow’s diet at 100 g/kg has beneficial effects on the fecal microbiota and antioxidant status, without penalizing other gestation parameters.

## 1. Introduction

The European Union (EU) is considered the world-leading producer of olives (*Olea europaea*) at more than 10 million tons/year (54.2% of world production), with Spain being the major producer at around 6 million tons/year [1]. Consequently, huge amounts of several residues derived from the olive oil industry are generated. In fact, 9.6 million tons/year of by-products are generated by the olive mill industry in the EU (olive pomace, leaves, and stones), and close to 11.8 million tons of biomass originating from the olive tree pruning procedure is produced additionally [2]. Among the olive by-products, those most frequently used to feed animals with positive results are olive pomace (alperujo), olive pulp (dried and sifted olive pomace), and defatted olive pulp (orujillo), which differ in their processing [3]. These kinds of olive by-products have been tested and found to be satisfactory in livestock species, such as broilers (*Gallus gallus*) [4] and pigs [5,6]. Olive pulp has been found to be one of the most interesting olive by-products for animal feed due to its composition: a high percentage of ether extracts (more than 70% unsaturated fatty acids), interesting bioactive compounds (polyphenols and triterpenes with antioxidant properties), and a low moisture content. However, its high lignin content and low crude protein levels limit its inclusion in livestock formulation diets [5]. Usually, the recommended inclusion levels of olive pulp in broiler and pig diets are close to 5%, although inclusion levels of up to 10% have been tested and proven satisfactory [7,8].

Regarding swine nutrition, by-products of the olive industry have been studied for their inclusion in growing–finishing pig diets [5,6]. Nevertheless, the information in the literature about their inclusion in the diet for gestating sows is scarce. Often, several problems, such as constipation and stereotyped behavior, are suffered by gestating sows due to feeding restrictions [9]. There is now a consensus that ensuring an adequate fiber intake in gestating sows can prevent constipation, in addition to increasing satiety and maintaining or even improving reproductive performance [9]. For this reason, high-fiber by-products, such as olive pulp, could potentially be used as ingredients in gestating sow diets.

In the traditional production system of Iberian pigs, the use of local resources for animal feed, such as olive pulp, offers an alternative to decrease the production cost and harmful environmental impact of farming, and it is particularly promising for gestating Iberian sows, which can benefit from its digestive properties. Thus, olive by-products can be considered a local source product, considering the proximity of the production and consumption places and the consequent reduction in environmental damage. Due to its fiber and bioactive compounds content and its potential as a circular economy strategy, the olive pulp effect should be tested in gestating sow diet. Thus, the hypothesis of this trial proposes the inclusion of olive pulp in Iberian sow diet without affecting the performance, metabolic, immunologic, and digestive status of sows, and their litter. The aim of this work was to assess the effects of olive pulp addition in multiparous Iberian sow diet at 100 g/kg in terms of their growth and litter performance, blood biochemical parameters and antioxidant status, and fecal microbiology.

## 2. Materials and Methods

### 2.1. Source of Olive Pulp

The olive pulp employed in this study was supplied by Dcoop Sociedad Cooperativa Andaluza, Antequera, Spain, and it came from dried, sieved, and pelleted olive pomace, which was treated to remove most of the stones, keeping an olive oil content of around 12%. The chemical composition analyzed (expressed on a dry matter basis) was: 8.2% for crude protein (CP), 12.6% for ether extract (EE), 9.9% for ash, 32.9% for neutral detergent fiber (NDF), 23.8% for acid detergent fiber (ADF), and 12.1% for acid detergent lignin (ADL). These analyses were carried out according to the methods described in the Section 2.5. The experimental diet was formulated considering a net energy (NE) level for olive pulp of 1.23 Mcal NE/kg indicated by the Spanish Foundation for the Development of Animal Nutrition [10].

### 2.2. Experimental Design, Animals, Facilities, and Diets

This research was performed on a pig farm in an extensive commercial production system owned by Dcoop Sociedad Cooperativa Andaluza, Antequera, Spain. A total of 40 purebred multiparous Iberian sows (149.6 ± 20.2 kg), which became pregnant after insemination with purebred Duroc boar semen, were randomly allocated to two different diets, with 20 sows in each treatment, located on collective parks. The production method was based on the traditional ‘camping system’, where the Iberian sows spend their gestation period in fenced outdoor facilities provided with metal shelters. Iberian sows were housed in pens (50 × 50 m), which had dirt floors and several trees, and each one had sufficient feeders and drinkers for 20 sows.

The two diets supplied were as follows: a control diet without olive pulp (CON) and an experimental diet with olive pulp at 100 g/kg (PUL). Both diets (Table 1) were isoenergetic and isoproteic, formulated in a meal form according to the requirements for gestating Iberian sows, as indicated by FEDNA [11]. The chemical composition and fatty acids profile of diets are shown in Table 2. The gestation status was confirmed at day 42 after insemination. During the gestation period, from insemination to one week before the planned farrowing day, all sows were fed following a restricted feeding program at a rate of 2 kg/animal per day (on a dry matter basis), and refused feed was not observed. One week before the expected farrowing day, all sows were moved from the camping facilities and were housed in individual farrowing boxes; the crates measured 2.4 m by 1.8 m and were equipped with a feeder, drinker, and climate-control system.

### 2.3. Growth and Litter Performance

The body weight (BW) of each sow was measured at post-insemination days 42 and 107, corresponding to gestation day 42 and the last gestation day before leaving the collective parks, respectively. Additionally, backfat thickness evaluation was recorded by using a linear probe (5.0 MHz transducer) connected to a B-mode ultrasound scan device (SF 1 Wireless Backfat and Loin Depth Scanner, Sonivet, Beijing, China). The scanning depth was programed at 45 mm, and measurement was taken at 65 mm from the midline at the last rib (P2 site). During the farrowing period, each sow was monitored, and the total litter weight and litter weight of piglets born alive were recorded, as well as the number of piglets born alive and the number of stillborn piglets.

### 2.4. Blood and Fecal Sample Collection

At 107 d gestation, 10 sows from each dietary group, with BW close to average weight, were chosen for feces and blood sample extraction. Fecal samples were obtained from the rectum of each animal, collected in sterile containers, and stored at −80 °C until analysis. Blood samples were obtained via ophthalmic venous sinus puncture using vacuum tubes of 5 mL without additives (Vacuette^®^, Greiner Bio-One International GmbH, Kremsmünster, Austria). Next, blood samples were centrifuged at 2000× *g* for 10 min to collect serum. Immediately, the serum was frozen at −20 °C until further analysis. In addition, 15 average-weight piglets of 10 days old, from sows belonging to each treatment, were randomly selected, and blood samples were obtained from them by jugular venipuncture into vacuum tubes (like those previously described). The processing for these samples was the same as that indicated for the sows’ blood.

### 2.5. Analytical Procedures

Olive pulp and diet samples were milled through a 1 mm screen (Retsch ZM 200, Retsch GmbH, Haan, Germany). For the DM, CP, EE, and ash contents, the Official Analytical Chemists Association procedures were followed [12]. The NDF, ADF, and ADL fractions were analyzed according to Van Soest et al. [13].

Complementary, starch, P, Ca, and fatty acid contents were analyzed in diet samples. Starch content determination was conducted by the official polarimetry technique according to the methods of Commission Regulation (EC) No. 152/2009 [14]. To analyze P and Ca concentrations, ash samples were diluted in 0.6 N HNO_3_ solutions and further filtered. According to the official analytical method indicated by Commission Regulation (EC) No. 152/2009 [14], the vanadate–molybdate method was used to assess P levels; and the analysis for determination of Ca was performed by an atomic absorption spectrophotometer (Unicam M series, Solar House, Thermo Fisher Scientific, Waltham, MA, USA). The fatty acid profile was analyzed using the UNE-EN ISO 5508:1996 [15] procedure by gas chromatography.

For the antioxidant status assessment of sows’ serum, three different spectrophotometric methods were carried out: cupric reducing antioxidant capacity (CUPRAC), Trolox equivalent antioxidant capacity (TEACH), and ferric reducing ability of plasma (FRAP) methods [16]. In all methods, the unit established to express the data was mmol Trolox equivalents/liter. Referring to the serum biochemical profile of Iberian sows, albumin, total protein, globulin, triglyceride, creatinine, urea, total cholesterol, and glucose were determined using an Olympus AU600 automated chemistry analyzer (Olympus Diagnostica Europe 166 GmbH, Ennis, Ireland). Serum immunoglobulins (Ig: IgG, IgM, and IgA) on day 107 of sow gestation and day 10 of piglet life were determined via specific enzyme-linked immunosorbent assay (ELISA) using a Pig IgG ELISA kit, Pig IgM ELISA kit, and Pig IgA ELISA kit (Bethyl Laboratories, Inc., Montgomery, TX, USA). All methods had an intra-assay and inter-assay imprecision lower than 15%.

Fecal bacteria counts were determined by real-time quantitative polymerase chain reaction (qPCR), employing the primer sequences and procedure described in Sánchez et al. [17]. The procedure was executed as follows: DNA extraction from fecal samples using a Favor Prep Stool Isolation Mini kit (Favorgen, Vienna, Austria); spectrophotometric quantification of DNA (Qubit 4 Fluorometer; Invitrogen, Thermo Fisher Scientific, Waltham, MA, USA); DNA amplification using the primers described; real-time PCR (qPCR) quantification assays using a CFX96 Real-time PCR Detection System thermocycler (Biorad Laboratories, Hercules, CA, USA); and standard curve generation and bacterial count determination (log copy number) by interpolating the values obtained.

### 2.6. Statistical Analyses

For all statistical analyses, the IBM SPSS Statistics system (IBM Corporation, Armonk, NY, USA) was used. The individual animal was taken as the experimental unit for all parameters assessed. For fecal microbiota data, the DNA copy numbers were logarithmically transformed (log_10_ DNA copy number) to meet normal distribution before statistical analysis. The Shapiro–Wilk test confirmed a normal distribution of all dependent variables, except for the number of stillborn piglets. Variance homogeneity for normal variables was verified using the Levene test. Thus, all parameters (performance, serum parameters, antioxidant capacity, Ig levels, and fecal microbiota data) were subjected to unpaired Student’s *t*-test, except for the number of stillborn piglets, which was analyzed using the Mann–Whitney U-test. The results are expressed as the mean ± SD. In all statistical analyses, differences were taken to be significant at *p* < 0.05.

## 3. Results

### 3.1. Growth and Litter Performance

According to the trial design, both diet intakes during the sow gestation period were similar. However, the number of stillborn piglets was lower (*p* = 0.013) in sows fed with olive pulp compared with that in control-diet-fed sows (Table 3). No differences were observed (*p* > 0.05) among dietary treatments in terms of sow BW and backfat thickness or in other litter parameters, such as total litter weight, litter weight of piglets born alive, and number of piglets born alive.

### 3.2. Serum Parameters and Antioxidant Capacity

The effects of olive pulp supplementation during gestation in the diet of Iberian sows on biochemical parameters and antioxidant capacity in serum are presented in Table 4. Albumin, total protein, triglyceride, creatinine, urea, and glucose levels were higher (*p* = 0.01, *p* = 0.007, *p* = 0.008, *p* = 0.004, *p* = 0.003, *p* = 0.040, respectively) in sows from the PUL group. The concentrations of globulin and total cholesterol in serum were not affected by dietary treatment (*p* > 0.05).

Dietary olive pulp inclusion during gestation enhanced the TEACH activity in sows’ serum compared with that in serum from sows fed the control diet (*p* = 0.013). Nevertheless, there were no differences (*p* > 0.05) at gestation day 107 regarding FRAP and CUPRAC activity in the serum of sows fed the control diet or olive-pulp-supplemented diet.

### 3.3. Ig Levels in Serum

IgG, IgM, and IgA serum immunoglobulin levels on gestation day 107 were not significantly (*p* > 0.05) different among dietary olive-pulp-supplemented sows and control-fed sows (Table 5). In the same way, piglets from sows fed an olive-pulp-supplemented diet showed IgG, IgM, and IgA serum levels at life day 10 that were similar to those in piglets from sows fed the control diet (*p* > 0.05).

### 3.4. Fecal Microbiota

The results showed an enhancement (*p* < 0.001) in *Lactobacillus* spp. and *Bifidobacterium* spp. fecal counts at 107 d in sows supplemented with olive pulp compared with those in sows fed the control diet (Table 6). Furthermore, an increase (*p* = 0.003) in the *Enterobacteriaceae* bacterial count was detected in the feces of sows fed with olive pulp. Nonetheless, olive pulp inclusion in the diets of sows during gestation had no effect on *Salmonella* spp. or *Clostridium* spp. among both groups (*p* > 0.05).

## 4. Discussion

Agro-industrial olive by-products have been targeted in studies aiming at their revalorization as low-value-added biomass (energy production), high-value-added biomass (pharmaceutical and cosmetic industries), and medium-value-added biomass (animal feed) [2]. Currently, in the scientific literature, there are no studies that consider olive by-product supplementation in the diet of gestating or lactating sows. However, the inclusion of olive by-products has been considered in growing–finishing pig feeding programs, with neutral or positive effects in terms of growth performance. The inclusion of partially defatted olive pulp [6] or olive pulp [8] in the diets of finishing pigs had no additional effect on animal performance when compared to conventional diets. In contrast, Liotta et al. [5] found that backfat thickness decreased linearly with increasing olive pulp level in the diet, being associated with a decrease in the energy content of the diet correlated to the increase in the fibrous fraction level. Generally, in swine nutrition, it is considered that dietary-fiber-rich ingredients do not promote growth performance or may even inhibit it owing to a decline in nutrient digestibility and energy deposition [18]. However, in sow nutrition restriction programs, given the existence of several problems related to constipation and stereotyped behavior, high-dietary-fiber diets play a relevant role in sow formulation programs. Based on the obtained results, the use of olive pulp at 100 g/kg in Iberian sow gestation diet did not result in worse growth or litter performance when compared with conventional ingredients. This effect was expected due to the diets being isoenergetic and isonitrogenous, and they were given on a restricted basis (2 kg/day; expressed on a DM basis) during the gestation period. However, we even detected a slight decrease in the number of stillborn piglets in sows fed the olive pulp diet. The effects of high-dietary-fiber ingredients’ (such as sugar beet pulp and soybean hulls) inclusion in sow diet on the number of stillborn piglets have been previously documented by Feyera et al. [19], who obtained similar results to those in this study, and proposed two mechanisms, which could be involved in this stillbirth rate reduction: sow constipation decreases caused by intestinal activity promotion and the elevated water-holding capacity of dietary fiber; and elongation of post-prandial energy uptake from the digestive tract, keeping stable glucose levels. Most studies carried out on farm animals considering olive by-products or olive extract supplementation have obtained variable effects on blood parameters related to either carbohydrates, fat, or protein metabolism. Despite glucose, creatine, and urea serum levels being higher in sows fed with olive pulp, it should be noted that the values found in this trial were close to the ranges previously reported in gestating sows [20,21]. An increase in albumin and total blood protein with olive pulp inclusion in diets has also been observed in broilers, and it is related to the enhanced antibody and immunity responses in animals [22]. Although in the current study albumin was increased, immunoglobulins were not affected in the serum of sows. Regarding triglycerides and total cholesterol levels when using olive pulp at 100 g/kg, the triglyceride concentrations were enhanced, but total cholesterol was not affected. The high oleic acid content in the PUL diet could explain the effect on the fat metabolism markers of sows fed with this diet. In this sense, the effect of oleic acid on pigs’ lipid metabolism has been documented, but the findings suggest differences between humans and pigs [23]. Along this line, Al-Harthi [7] tested an olive by-product in broiler diet and obtained similar effects to those obtained in this trial in terms of a triglyceride level increment; however, in his case, the total cholesterol concentration was positively affected, probably due to the unsaturated and polyunsaturated fatty acid content of olive pulp. On the other hand, Paiva-Martins et al. [24] did not show an effect on the concentration of triglycerides and other fatty metabolism parameters when olive leaves were included in growing pigs’ diet. These differences between by-products are possibly due to the lower fat content of olive leaves.

It is widely known that gestation and lactation in sows entail an increased metabolic burden, resulting in systemic oxidative stress and fading antioxidant capacity. Additionally, insufficient average daily feed intake during lactation, as one of the main complications limiting sow reproductive performance, is partially produced by disproportionate oxidant and antioxidant status [9]. In this context, by-products rich in fiber and antioxidant compounds arise as a promising alternative in sow feed. It should be noted that olive derivatives are one of the by-products richest in fiber and antioxidant compounds. Functional compounds, such as hydroxytyrosol, tyrosol, cinnamic acid, β-carotene, oleuropein, and monounsaturated and polyunsaturated fatty acids, are abundantly present in them [25]. In addition to this, a correlation between sow dietary fiber composition during gestation and variations in the antioxidant activity of plasma and different tissues has already been proposed in the literature [26]. According to the current results, olive pulp supplementation in Iberian sow gestation diet enhanced blood TEACH activity compared with that in Iberian sows fed a diet with no supplement. Nevertheless, no differences were observed in the FRAP or CUPRAC blood activity between the sow groups. The capacity of the antioxidant biocompounds present in olive products to scavenge free radicals has been amply demonstrated in humans by the consumption of olive oil [27]. In pigs, positive results have been obtained by assessing the rise in antioxidant status with oleuropein supplementation in pigs. Metal-chelating power, measured as the FRAP activity of blood, in pigs fed an oleuropein diet was significantly higher than that in pigs fed a standard diet [28]. In this study, no differences were observed in the FRAP activity of Iberian sows’ blood. It should be considered that the polyphenols’ antioxidant power depends on the extent of hydroxylation and conjugation, and this can vary based on the kind of polyphenol [29].

The IgG, IgM, and IgA blood levels in sows at gestation day 107, and the Ig levels in piglets at 10 days of life, were not influenced by the olive pulp dietary treatment. Both sow and piglet immunoglobulin blood concentrations were in concordance with the levels reported in the literature [30,31]. Blood Ig level variations depending on the fiber source have been studied before, and the results were similar to this study. Shang et al. [32] tested two sources of fiber (sugar beet pulp and wheat bran) in sow late gestation and lactation diets, and the IgG, IgM, and IgA serum levels were not affected by the included fiber source. Nonetheless, other sources of polyphenolic compounds, such as grape seed extracts, have been proposed to improve the immunoglobulin response in animals [33].

Iberian sows fed olive pulp showed an increase in the fecal contents of *Lactobacillus* spp. and *Bifidobacterium* spp. Both polyphenolic compounds and fiber content or type, soluble or insoluble, included in the diet can affect the intestinal microbiota composition of animals [34,35]. Regarding fiber type, there is rising evidence for a helpful growth upgrade, such as lactobacilli and bifidobacteria in the small intestine of pigs, through inclusion of some dietary fiber types, such as insoluble fiber [36]. Lactobacilli and bifidobacteria are well-known probiotics, with multiple health-promoting effects, such as suppression of gut inflammation, improvement of intestinal barrier function, modulation of immune responses, microbial homeostasis, and prevention of disease bacteria [32]. However, the effects of the inclusion of olive pulp and other olive by-products (products rich in insoluble fiber) in livestock diets on the gut microbiota have barely been tested. Several authors have suggested that some modified olive oil by-products, such as ‘alperujo’, included in poultry diets can modulate the intestinal microbiota, increasing or reducing certain phyla, families, or genera of gut bacteria. Along this line, Rebollada-Merino et al. [4], by adding fermented defatted ‘alperujo’ (FDA) to laying hens’ diet and comparing with laying hens fed a conventional diet, found that hens fed a diet with FDA had greater abundance of Actinobacteria, Firmicutes, and Proteobacteria and higher bacterial diversity. These results are partially in agreement with those of the current study, since Actinobacteria and Firmicutes are the phyla to which *Bifidobacterium* spp. and *Lactobacillus* spp. belong, respectively. The proliferation of these bacteria may improve host digestive health by producing metabolites, enhancing immune status, and displacing harmful bacteria. On the other hand, Ferrer et al. [6], who tested partially defatted olive pulp inclusion (120 g/kg) in finishing pig diets and its influence on the fecal microbiota, found no effect on *Lactobacillus* spp., *Bifidobacterium* spp., and *Enterobacteriaceae* values. The variable effects of supplementation with olive by-products in livestock diets in terms of intestinal microbiota observed in the scientific literature are probably due to the high differences in the chemical composition of olive by-products, or even due to bacterial adaptation to olive pulp presence or limitations of culture-based techniques [3]. In addition, the enhancement in sows’ gut of *Lactobacillus* spp. and *Bifidobacterium* spp. populations by olive pulp inclusion is important even to the offspring, since the piglets’ intestinal tract colonization is started at birth by transmission from the maternal microbiota, along with other sources [37].

## 5. Conclusions

In conclusion, olive pulp inclusion at 100 g/kg in a feed formulation for Iberian sows during their gestation period can be considered, as it did not negatively affect growth or litter performance, and it even provided antioxidant status and gut health improvements. In addition, from an environmental and profitability viewpoint, this kind of local source by-product represents an interesting alternative to reduce feed costs in the Iberian swine production industry under a circular economy approach.

## Figures and Tables

**Table 1:** Diet composition (g/kg) and nutrient levels estimates of diets (g/kg, as-fed basis) for Iberian gestating sows.

Raw Material	Diets	
	CON	PUL
Barley	309.30	305.60
Wheat	286.70	300.00
Wheat bran	250.00	80.00
Olive pulp	-	100.00
Canola	70.50	73.30
Sunflower meal 26%	44.50	60.00
Soybean 47%	-	29.80
Lard	10.00	23.70
Calcium carbonate	13.40	9.80
Salt	5.00	4.90
Premix ^1^	5.00	5.00
Dicalcium phosphate	1.60	3.90
L-Lysine	2.00	2.00
Mycosorb ^2^	2.00	2.00
Calculated nutrient composition (g/kg, as-fed basis) ^3^		
CP (*N* × 6.25)	138.00	138.00
NDF	227.86	214.94
NE, Mcal/kg	2.20	2.20
SID lysine	5.00	5.00

Abbreviations: CON = control diet; PUL = diet with inclusion of 100 g/kg of olive pulp; CP = Crude Protein; NDF = Neutral Detergent Fiber; NE = net energy; SID lysine = Standardized Ileal Digestible lysine. ^1^ Premix provided vitamins and minerals per kg CON and PUL diets as follows: vitamin A (3a672a), 9000 IU; vitamin D3 (3a671), 2000 IU; vitamin E (3a700), 30 IU; vitamin K3 (3a711), 2 mg; vitamin B1 (3a821), 2 mg; vitamin B2 (3a825ii), 4 mg; vitamin B6 (3a831), 1.5 mg; vitamin B12, 0.015 mg; niacin (3a315), 15 mg; pantothenic acid (3a841), 16.75 mg; folic acid (3a316), 2 mg; biotin (3a880), 0.2 mg; Mn (3b502), 30 mg; Zn (3b603), 110 mg; Cu (3b405), 10 mg; I (3b201), 0.55 mg; Se (3b802), 0.2 mg; Fe (3b103), 72 mg; 6-phytase (EC 3.1.3.26), 500 FTU; sepiolite (E562), 585 mg; butylated hydroxyanisole (E320), 1.575 mg; butylated hydroxytoluene (E321), 7.875 mg. ^2^ Mycosorb^®^ Alltech Inc., Nicholasville, KY, USA. ^3^ According to Spanish Foundation for the Development of Animal Nutrition (FEDNA, 2019).

**Table 2:** Analyzed nutrient composition (g/kg, on a dry matter basis) and fatty acids profile (g/kg of fat) of diets for Iberian sows.

Item	Diets	
	CON	PUL
DM	906.07	904.63
CP	149.00	153.00
Ether extract	29.83	40.42
Total Starch	472.20	422.92
NDF	314.68	283.25
ADF	72.60	90.24
ADL	16.19	23.46
Ash	60.73	65.14
Ca	7.60	9.40
P (total)	4.50	4.30
Fatty acids composition		
Capric acid [C10]	0.50	0.50
Lauric acid [C12]	0.80	0.90
Myristic acid [C14]	6.20	6.60
Palmitic acid [C16]	210.30	205.70
Palmitoleic acid [C16:1]	10.70	13.70
Stearic acid [C18]	59.00	67.90
Oleic acid [C18:1]	314.00	407.00
Linoleic acid [C18:2]	365.00	268.00
Linolenic acid [C18:3]	27.30	24.00
Arachidic acid [C20]	5.90	5.40
Arachidonic acid [C20:4]	0.70	0.70

Abbreviations: CON = control diet; PUL = diet with inclusion of 100 g/kg of olive pulp; DM = Dry Matter; CP = Crude Protein; NDF = Neutral Detergent Fiber; ADF = Acid Detergent Fiber; ADL = Acid Detergent Lignin.

**Table 3:** Effects of olive pulp dietary inclusion on growth and litter performance of Iberian sows (mean ± SD).

Item	CON	PUL	*p*-Value
Sample size	20	20	
Body weight (kg)			
Day 42	153.10 ± 17.115	146.00 ± 22.886	0.278
Day 107	185.13 ± 20.422	184.00 ± 23.582	0.881
Backfat thickness (mm)			
Day 42	28.45 ± 6.807	24.98 ± 7.639	0.119
Day 107	29.61 ± 5.495	27.46 ± 7.256	0.269
Litter performance			
Total litter weight (kg)	12.86 ± 3.045	11.80 ± 2.385	0.226
Litter weight of piglets born alive (kg)	11.78 ± 2.596	11.64 ± 2.316	0.853
Number of piglets born alive	8.09 ± 1.975	7.58 ± 1.644	0.377
Number of stillborn piglets ^1^	1.17 ± 1.403	0.26 ± 0.562	0.013

Abbreviations: CON = control diet; PUL = diet with inclusion of 100 g/kg of olive pulp. ^1^ The presented *p*-value is based on Mann–Whitney U-test.

**Table 4:** Serum parameters and antioxidant capacity of Iberian sows fed control and olive-pulp-supplemented diet during gestation period (mean ± SD).

Item	CON	PUL	*p*-Value
Sample size	10	10	
Serum parameters			
Albumin (g/dL)	3.65 ± 0.211	4.13 ± 0.228	<0.001
Total protein (g/dL)	7.32 ± 0.581	8.01 ± 0.395	0.007
Globulin (g/dL)	3.67 ± 0.570	3.86 ± 0.374	0.374
Triglyceride (mg/dL)	67.55 ± 43.696	124.90 ± 38.565	0.008
Creatinine (mg/dL)	1.14 ± 0.176	1.37 ± 0.125	0.004
Urea (mg/dL)	19.91 ± 7.307	32.61 ± 8.001	0.003
Total cholesterol (mg/dL)	88.12 ± 25.324	74.58 ± 13.459	0.148
Glucose (mg/dL)	63.85 ± 7.811	75.31 ± 12.900	0.040
Antioxidant capacity			
TEACH ^1^ (mmol/L)	0.37 ± 0.031	0.42 ± 0.039	0.013
FRAP ^2^ (mmol/L)	0.32 ± 0.100	0.34 ± 0.055	0.499
CUPRAC ^3^ (mmol/L)	0.35 ± 0.041	0.38 ± 0.018	0.150

Abbreviations: CON = control diet; PUL = diet with inclusion of 100 g/kg of olive pulp. ^1,2,3^ Trolox equivalent antioxidant capacity, ferric reducing ability of plasma, and cupric reducing antioxidant capacity, respectively.

**Table 5:** Effect of dietary inclusion of olive pulp on IgG, IgA, and IgM levels (mg/mL) in serum of Iberian sows and piglets (mean ± SD).

Groups	CON	PUL	*p*-Value
Sows’ Serum ^1^			
Sample size	10	10	
IgG	48.50 ± 10.213	47.75 ± 11.389	0.889
IgA	1.39 ± 0.974	1.50 ± 0.695	0.800
IgM	6.09 ± 2.119	6.12 ± 1.245	0.971
Piglets’ Serum ^2^			
Sample size	15	15	
IgG	35.03 ± 11.749	36.93 ± 10.662	0.675
IgA	0.94 ± 0.673	0.98 ± 0.613	0.877
IgM	0.50 ± 0.375	0.48 ± 0.325	0.923

Abbreviations: CON = control diet; PUL = diet with inclusion of 100 g/kg of olive pulp; Ig = immunoglobulin. ^1^ Sows’ serum samples collected at gestation day 107. ^2^ Piglets’ serum samples collected at life day 10.

**Table 6:** Effect of olive pulp dietary addition on the fecal contents of *Enterobacteriaceae*, *Salmonella* spp., *Clostridium* spp., *Lactobacillus* spp., and *Bifidobacterium* spp. (log_10_ number of copies DNA/g feces) in Iberian sows (mean ± SD).

Taxonomic Rank	CON	PUL	*p*-Value
Sample size	10	10	
*Enterobacteriaceae*	6.80 ± 0.046	6.86 ± 0.031	0.003
*Salmonella* spp.	4.72 ± 0.111	4.60 ± 0.159	0.107
*Clostridium* spp.	9.12 ± 0.345	9.15 ± 0.509	0.890
*Lactobacillus* spp.	8.37 ± 0.175	9.05 ± 0.110	<0.001
*Bifidobacterium* spp.	7.38 ± 0.144	8.43 ± 0.205	<0.001

Abbreviations: CON = control diet; PUL = diet with inclusion of 100 g/kg of olive pulp.

## Data Availability

None of the data were deposited in an official repository. Data that support the study findings are available upon request.
